# Advances in Therapeutic Targeting of Cancer Stem Cells within the Tumor Microenvironment: An Updated Review

**DOI:** 10.3390/cells9081896

**Published:** 2020-08-13

**Authors:** Kevin Dzobo, Dimakatso Alice Senthebane, Chelene Ganz, Nicholas Ekow Thomford, Ambroise Wonkam, Collet Dandara

**Affiliations:** 1 International Centre for Genetic Engineering and Biotechnology (ICGEB), Cape Town Component, Wernher and Beit Building (South), UCT Medical Campus, Anzio Road, Observatory, Cape Town 7925, South Africa; dimakatsosenthebane@gmail.com (D.A.S.); cheleneganz@gmail.com (C.G.); 2 Division of Medical Biochemistry and Institute of Infectious Disease and Molecular Medicine, Department of Integrative Biomedical Sciences, Faculty of Health Sciences, University of Cape Town, Cape Town 7925, South Africa; 3 Division of Human Genetics, Department of Pathology and Institute for Infectious Disease and Molecular Medicine, Faculty of Health Sciences, University of Cape Town, Anzio Road, Observatory, Cape Town 7925, South Africa; n.e.thomford@uccsms.edu.gh (N.E.T.); Ambroise.wonkam@uct.ac.za (A.W.); collet.dandara@uct.ac.za (C.D.); 4 Department of Medical Biochemistry, School of Medical Sciences, College of Health Sciences, University of Cape Coast, PMB, Cape Coast, Ghana

**Keywords:** cancer stem cells, tumor microenvironment, metastasis, drug resistance, ABC transporters, epithelial to mesenchymal transition, hypoxia, clinical trials

## Abstract

Despite great strides being achieved in improving cancer patients’ outcomes through better therapies and combinatorial treatment, several hurdles still remain due to therapy resistance, cancer recurrence and metastasis. Drug resistance culminating in relapse continues to be associated with fatal disease. The cancer stem cell theory posits that tumors are driven by specialized cancer cells called cancer stem cells (CSCs). CSCs are a subpopulation of cancer cells known to be resistant to therapy and cause metastasis. Whilst the debate on whether CSCs are the origins of the primary tumor rages on, CSCs have been further characterized in many cancers with data illustrating that CSCs display great abilities to self-renew, resist therapies due to enhanced epithelial to mesenchymal (EMT) properties, enhanced expression of ATP-binding cassette (ABC) membrane transporters, activation of several survival signaling pathways and increased immune evasion as well as DNA repair mechanisms. CSCs also display great heterogeneity with the consequential lack of specific CSC markers presenting a great challenge to their targeting. In this updated review we revisit CSCs within the tumor microenvironment (TME) and present novel treatment strategies targeting CSCs. These promising strategies include targeting CSCs-specific properties using small molecule inhibitors, immunotherapy, microRNA mediated inhibitors, epigenetic methods as well as targeting CSC niche-microenvironmental factors and differentiation. Lastly, we present recent clinical trials undertaken to try to turn the tide against cancer by targeting CSC-associated drug resistance and metastasis.

## 1. Introduction

Cancer remains one of the major causes of mortality globally, with many recent studies showing significant increases in its incidence [1,2]. Recent advances in cancer diagnosis and treatment have resulted in improvements in patients’ outcomes, however, several hurdles remain including drug resistance, cancer relapse and metastasis [3]. Drug resistance which can lead to relapse continues to be associated with fatal disease [3]. Data from several studies reveal that therapy resistance and chemoresistance in particular limits the therapeutic value of many drugs, resulting in relapse and metastasis [4]. Senthebane and colleagues revealed that tumor microenvironment (TME) components including cancer-associated fibroblasts (CAFs) and the extracellular matrix (ECM) are major contributors to chemoresistance [3]. Recent data also points to cancer stem cells (CSCs) as responsible for therapy resistance and metastasis [5,6,7].

CSCs have been defined as a subset of cancer cells with the ability to self-renew and to differentiate into non-CSC cancer cells within the tumor mass [6,8]. The CSC field was shaped by great research done on hematopoietic stem cells (HSCs). HSCs are hierarchically arranged with HSCs being the founder cells that undergo asymmetric cell division giving rise to differentiated daughter cells and one quiescent stem cell with self-renewal abilities [9]. The dividing daughter cells will over time become restricted in terms of lineages it can form. The studies on HSCs ignited research on mammalian tissue and cell renewal as well as in cancer. In addition, cancer patients with chronic myeloid leukemia (CML) were shown to have rare quiescent cells also referred to as Philadelphia chromosome-positive and BCR-ABL-positive cells and these cells were able to resist drug treatment [10,11]. The above-mentioned studies and revelations allowed further research on self-renewal and eventually gave birth to the CSC field as it is today. CSCs are able to reproduce primary tumor heterogeneity as well as metastases in distant tissues and organs [12]. As postulated by Paget, cancer cells can escape the primary tumor site and spread to other tissues and organs where they can proliferate and therefore act as “seeds” for the growth of secondary tumors [12]. It is possible that cancer cells can detach from the primary tumor and enter circulation, however, they are likely not to survive the arduous journey to other organs and cannot “seed” metastases at secondary sites. With their demonstrable survival abilities, enhanced expression of transmembrane transporters and tumorigenic abilities, CSCs on the other hand are likely to survive in circulation and be able to “seed” new tumors at secondary sites [13,14]. CSCs are also responsible for the development of therapy resistance, with many studies demonstrating that CSCs are able to withstand conventional therapies such as chemotherapy and radiotherapy [15]. The ability to resist conventional therapies has been attributed to many properties including increased expression of drug transporters, maintenance of a slow dividing state (quiescence) as well as efficient DNA repair mechanisms [16,17,18]. To overcome CSC resistance, new therapies are under development including epigenetic therapies, immunotherapy as well as drugs targeting angiogenesis [19].

From the early days of their discovery, many studies have shown that CSCs are undifferentiated tumor cells able to generate tumors [20,21,22]. To date, several studies have been able to prove the existence of CSCs in cancers such as CML, ovarian, lung and breast cancer [23,24]. Methods used to identify CSCs range from antibody-based isolation, enzyme activity of ALDH, tumorsphere formation, use of dyes such as PKH26 and side population sorting [25,26]. Side population cells display enhanced abilities to efflux dyes and drugs at a higher rate than the main cell population due to increased expression of ATP-binding cassette (ABC) transporter proteins. These methods are all not specific, and in most cases, scientists combine these methods to get a cell population with high numbers of CSCs. The gold standard method to study whether cancer cells have tumor-initiating capabilities is the use of limiting dilution in xenograft animals. A detailed review of CSCs definition and terminology is provided by Valent and colleagues [15]. Recently introduced “humanized” animal models are better models than traditional animal models as they can recapitulate some human cancers better [27,28].

**Figure 1:**
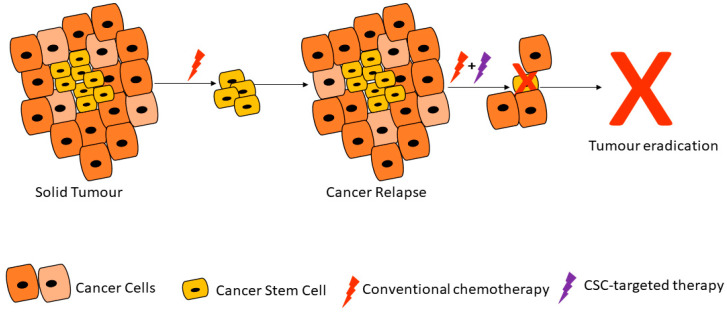
Cancer stem cells are able to resist conventional therapies and form new tumors, unless targeted by cancer stem cell (CSC)-specific therapy. Adapted from Dzobo et al. [30].

Due to their ability to resist therapy, CSCs can travel to distant sites and form new tumors. Whilst the process of metastasis appears disorganized, metastatic lesions are the main cause of cancer deaths and therapy resistance [29]. Signaling pathways upregulated and dysregulated in CSCs and CSC-cell interactions are therefore some of the targets of new drugs under development. Conventional cancer treatment strategies mainly target rapidly proliferating cancer cells and can reduce tumor mass, tumor relapse can result from a few remaining cancer cells including CSCs (Figure 1) [30]. Our ability to target CSCs largely depends on new evidence and in-depth characterization of these cells. It is plausible to postulate that long-lasting cancer treatment efficacy can only come from both shrinkage of the primary tumor as well as the prevention of cells such as CSCs from metastasizing to new sites throughout the body.

This review is an updated critical analysis and distillation of available information on CSCs and their involvement in cancer therapy resistance and metastasis. By targeting inherent CSCs properties that allow CSCs to be tumorigenic, resistant and also metastatic, new drugs being developed offer a better promise at curing cancer.

## 2. Properties of Cancer Stem Cells

### 2.1. Cancer Stem Cell Markers and Therapy Resistance

**Figure 2:**
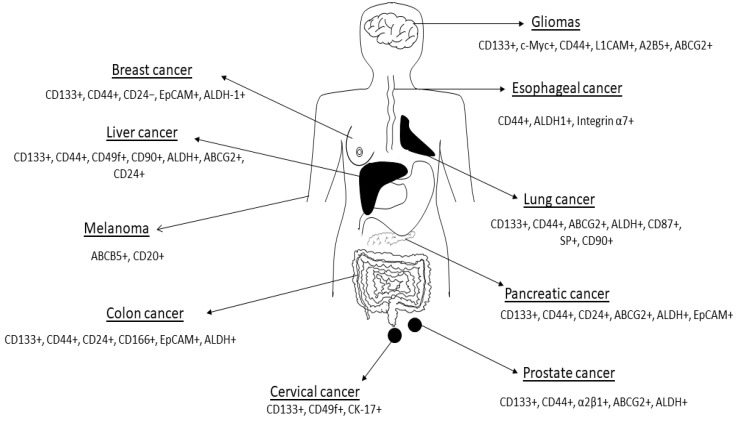
Cancer stem cell markers expressed in some human cancers are shown in the figure. Figure adapted from Dzobo et al. [34]. See Table 1 for references. The list of CSC markers is not exhaustive. The CSC markers continue to be refined based on new data.

Current therapies are unable to eliminate cancer partly due to CSCs’ enhanced ability to withstand treatment regimens [15,30]. CSCs are thought to account for a small percentage of the total number of cancer cells within a tumor but have self-renewal and differentiation capabilities [21]. A major hurdle faced by scientists working with CSCs has been the isolation and characterization of these cells. Antibodies against several CSC markers have been used to isolate CSCs from solid tumors [26]. Commonly used CSC markers and methods for isolation and characterization include CD24, CD44, CD133 and ALDH enzymatic assay (Figure 2; Table 1) [31]. These CSC markers are either used alone or in different combinations in different cancers. For example, gastric CSCs display high CD44, CD133 as well as Lgr5 [32]. Lung CSCs express several markers including CD133+, ALDH1+ and CD44+ [33]. Whilst the same CSC markers can be found in different cancers, some cancers have distinct markers for example melanoma CSCs are ABCB5+ whilst medulloblastoma CSCs are CD15+ (Table 1).

Several studies demonstrated an increase in CSCs in tumors after cancer treatment, clearly illustrating their persistence during treatment [75,76,77]. CSCs are able to resist therapeutic interventions due to several reasons including their cellular plasticity, enhanced expression of ABC drug transporters, ability to detoxify of drugs and compounds, increased adaptation to stressful conditions such as hypoxia, attaining quiescence and activation of survival pathways [77,78,79].

CSCs ability to resist therapy is widespread and referred to as multidrug resistance. This capability stems from the ability of CSCs to express increased detoxifying enzymes, increased activation of survival signaling pathways, DNA repair mechanisms as well as drug efflux pumps [30,78]. In addition, CSCs have been noted for their immune evasion capabilities, their ability to undergo epithelial to EMT as well as to adapt their metabolism to survive low nutrient conditions [77,78]. Thus, the hallmarks of CSCs include quiescence, increased expression of drug metabolizing and detoxifying enzymes, enhanced DNA reparability, the ability to undergo EMT and overexpression of ABC membrane transporters. Lately, CSCs have also been shown to undergo epigenetic reprogramming, making them very difficult to eradicate in cancers [80].

The ALDH superfamily is a large family of proteins and several members including aldehyde dehydrogenase 1 (ALDH1) have been implicated in drug detoxifying activities [81,82]. In its entirety, the ALDH superfamily is composed of 19 enzymes with ALDH1 being the main isoform [81,82,83]. This family of detoxifying enzymes is involved in the oxidation of aldehydes to carboxylic acids as well as retinol to retinoic acid [84,85]. Besides being expressed by normal cells, ALDH1 is expressed highly in CSCs [86,87]. As a result, ALDH1 expression and activity can be used reliably to identify CSCs in some cancers. Vogler and colleagues demonstrated that ALDH1 expression can be used as an independent prognostic marker for low survival in colorectal patients [88]. In addition, van den Hoogen and coworkers also showed that enhanced ALDH1 activity can be used to identify tumor-forming cells as well as cells with the propensity to form prostate cancer metastases [89]. Ueda and colleagues also showed that ALDH1 activity can be used to identify cancer cells with CSC-like properties in human renal cell carcinoma cell line [90]. Ginestier and colleagues demonstrated that ALDH1 is highly expressed in breast CSCs and is a predictor of poor clinical outcome [91]. In addition, ALDH1-expressing cells were able to form xenograft tumors easily [91]. Several other studies demonstrated the successful transplantation of ALDH1-expressing cells into mice [92,93]. The expression of ALDH1 by normal stem cells may explain the aberrant expression of this enzyme in CSCs as normal stem cells are a potential source of CSCs, among other cells [94]. Furthermore, ALDH1 expression has been shown to allow CSCs to resist conventional therapy including commonly used drugs such as paclitaxel, gemcitabine and cisplatin [95,96]. In agreement with the above, several studies demonstrated that inhibition of ALDH1 activity in CSCs sensitizes these cells to several drugs, linking ALDH1 with therapy resistance [97,98].

**Figure 3:**
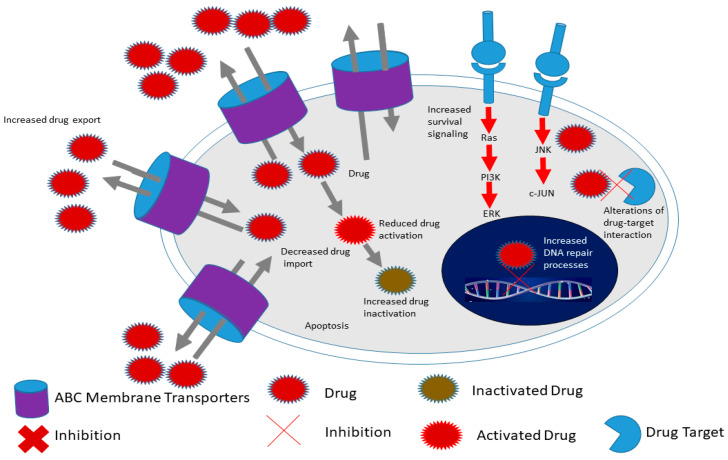
Hallmarks of cancer stem cells include increased expression of ATP-binding cassette (ABC) membrane transporters, enhanced survival signaling, increased drug in activation as well as increased DNA repair processes compared to cancer cells. This allows CSCs to survive conventional therapy and thus contribute to chemoresistance for example. Adapted from Senthebane et al. [3].

In addition, CSCs demonstrate increased expression of drug effluxing proteins such as the ABC transporters (Figure 3) [99,100,101]. The ABC family of transporters consists of 49 molecules using ATP as an energy source during the trafficking of proteins across the cell membrane. Many studies have been performed on the characterization of members of this family including ABCB1 (multidrug resistance 1 (MDR1)), ABCG2, ABCC1 and ABCB5 [102,103]. Through elaborate experiments, several research groups demonstrated that CSCs aberrantly express ABC transporters and are able to withstand toxic levels of drugs and other toxins [104,105]. In elaborate experiments performed by Wright and colleagues, the researchers demonstrated that ABCB1 was aberrantly overexpressed in breast CSCs causing resistance to conventional chemotherapy such as paclitaxel and doxorubicin [106]. Frank and coworkers demonstrated that ABCB5 was overexpressed and caused resistance to doxorubicin in CD133+ circulating melanoma cells [107]. Through the use of a monoclonal antibody against ABCB5, the authors were able to induce cancer cell sensitivity to drugs such as doxorubicin [107]. Shi and colleagues demonstrated that ABCG2-expressing CSCs isolated from hepatocellular carcinoma cell lines via the side population technique are able to resist cisplatin and 5-fluorouracil [108]. The above studies and others demonstrated that inhibition of ABC transporters is a potential mechanism of overcoming CSC chemoresistance [109,110]. Several studies have been performed on the inhibition of ABC transporters and have shown remarkable success in sensitizing both cancer cells and CSCs to several drugs [111,112]. For example, Marcelletti and colleagues utilized zosuquidar, an inhibitor of P-gp (ABCB1) to sensitize cancer cells in acute myeloid leukemia [111,112].

Several other proteins associated with apoptosis are also involved in the survival of cancer cells and CSCs [113,114]. For example, several pro-survival proteins including BCL-2, B-cell lymphoma extra-large (Bcl-xL) and BCL-2-like-2 (BCL-W) have been found to be overexpressed in several cancer types including lymphoid cancer [115,116]. The overexpression of these pro-survival proteins has also been linked with carcinogenesis, with the blocking of these proteins and their associated pathways resulting in reduced tumor growth and enhanced response to chemotherapy [116,117,118].

Cytotoxic drugs target rapidly growing cancer cells, making them ineffective against slow dividing or dormant CSCs [30]. Viale and colleagues demonstrated that leukemia CSCs proliferate at a much lower rate than other cancer cells [119]. Therapies that target cancer cell cycling would therefore be ineffective against CSCs. Therapeutic agents such as paclitaxel would be unable to be less effective against slow dividing CSCs [120]. In addition, several studies demonstrate that CSCs show enhanced DNA damage repair capacity, with phosphorylation of repair enzymes observed in cancers such as breast and gliomas [121,122]. CSCs including glioma stem cells demonstrate great abilities at ROS scavenging thus protecting themselves against oxidative DNA damage [123,124,125]. Therapy itself has been shown to selectively increase CSCs in tumors. For example, Rizzo and colleagues demonstrated that CSCs are enriched in ovarian tumors after chemotherapy [126]. In addition, Levina and coworkers showed that chemotherapy can lead to the propagation of CSCs in lung cancer [127]. Thus, chemotherapy only targets the rapidly proliferating cancer cells leaving the CSCs to propagate the tumor after therapy. Chen and colleagues demonstrated that the drug temozolomide (TMZ) activates CSCs to produce cancer cells after therapy [128]. Qiu and colleagues demonstrated that elevated O^6^-methylguanine DNA methyltransferase (MGMT) expression and activity in glioma stem-like cells were responsible for temozolomide resistance [129]. Kurtova and coworkers also demonstrated that blockage of tumor repopulation by CSCs is effective at attenuating therapy resistance in bladder cancer [130]. Saito and colleagues demonstrated that inducing cell cycle re-entry through treatment with granulocyte colony-stimulating factor (G-CSF) allows normal chemotherapy to eliminate cancer cells effectively [131]. In addition, the induction of CSCs differentiation has been used successfully to increase CSCs sensitivity to commonly used cancer drugs. Lombardo and coworkers induced colorectal CSCs terminal differentiation via the use of bone morphogenic protein 4 (BMP4) and observed increased CSCs sensitization to standard chemotherapy [132]. Wang and coworkers used silibinin, which blocks colon CSCs self-renewal, resulting in reduced CSC population leading to reduced cancer cell proliferation [133]. Whilst several strategies have been developed to induce CSCs differentiation, all-trans retinoic acid (ATRA) is one of the common drugs used for this purpose [134,135].

### 2.2. Cancer Stem Cells and Angiogenesis

**Figure 4:**
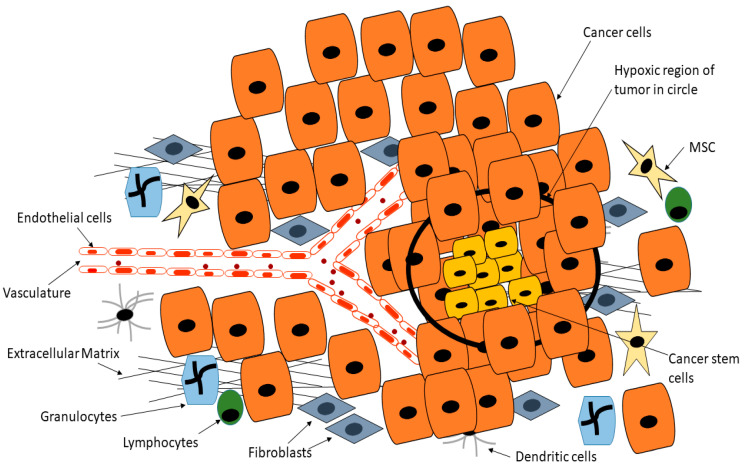
Cancer stem cells are able to reside deep within the tumor in hypoxic regions that are normally toxic to normal cells, whilst CSCs are able to release factors such as hypoxia-inducible factor 1 which induces the release of proangiogenic factors, this position means CSCs are inaccessible to drugs or are exposed to reduced drug doses. Adapted from Senthebane et al. [3].

Many biological processes are dependent on the formation of new blood vessels, a process referred to as angiogenesis. Normal development and tissue repair and regeneration are especially dependent on new blood vessels for the supply of nutrients as well as the removal of toxic material [136,137]. Besides normal biological activities, angiogenesis is a requirement for tumor formation beyond a certain diameter [138,139]. During tumor formation, the usual delicate balance between pro-angiogenesis and anti-angiogenesis is altered, with pro-angiogenesis factors dominating [138]. New blood vessels sprout from pre-existing vessels within and around the tumor, fueling the rapid growth of the tumor [140,141]. The rapid growth of a tumor results in hypoxic conditions within the tumor. CSCs are known to release factors such as hypoxia-inducible factor 1 which induces the release of proangiogenic factors (Figure 4) [142,143]. Hypoxia has also been shown to fuel CSCs [144]. Hypoxia promotes CSC growth in several cancers via the upregulation of adaptive transcriptional programs, allowing CSCs to survive, invade and metastasize [144,145]. Soeda and colleagues demonstrated that hypoxia promotes the self-renewal capacity of CD133-positive human glioma-derived cancer stem cells [144]. Heddleston and colleagues showed that hypoxia promotes CSC self-renewal capabilities and stem-like phenotype even in non-stem cancer cell populations [146]. As reviewed by Heddleston and colleagues, CSC plasticity is influenced by hypoxia and new drugs must target such microenvironmental conditions for durable cancer treatment [147].

Endothelial cells are also recruited to the tumor site. Endothelial cells express VEGFR and the binding of VEGF-A results in the activation of several signaling cascades involved in migration and ECM remodeling [143,148]. Survival pathways including the PI3K-Akt and the MEK-ERK cascades are activated and play key roles in the activation of endothelial cells to form new blood vessels [148]. Several cytokines are also known to be secreted by CSCs within the TME and these include IL-6 and TNF-α [149,150]. The secreted cytokines are involved in the recruitment of immune cells such as myeloid cells to further promote tumorigenesis [149,150]. Dysregulation of the Notch pathway has also been associated with tumor growth in general and survival of CSCs [151,152,153]. Several reports demonstrated that cells showing high expression of Notch signaling have elevated tumor-forming abilities and self-renewal capacity than those with less Notch activation [154,155,156]. Activation of the Notch pathway has also been associated with proangiogenic activity, with Notch ligand Jagged-1 promoting blood vessel formation [157,158,159].

In addition, matrix metalloproteases (MMPs) secreted by both cancer cells and stromal cells remodel the TME, allowing the creation of space for blood vessels formation as well as recruitment of different cells [160,161]. Due to the plasticity of CSCs, suggestions have been made to the effect that CSCs can give rise to endothelial cells and pericytes within and around the tumor [162,163]. Blood vessels within the TME are convoluted and “leaky”, resulting in fewer drugs able to reach cancer cells and CSCs deep within the TME. Tumor-derived cells are also able to intravasate and travel to distance sites, promoting metastasis in the process. Several studies have demonstrated the presence of circulating tumor-derived cells that are able to act as “seeds” for new tumors in distant sites [164,165]. Once the circulating cancer cells reach distant sites, they are able to extravasate and form new tumors in favorable microenvironments [19,166]. The formation of new tumors is dependent on CSCs successfully inducing angiogenesis to allow the exchange of nutrients and metabolic byproducts. Whilst it has been shown that stromal cells play a key role in inducing angiogenesis within tumors, CSCs are also involved in releasing angiogenic factors [167]. For example, Bao and colleagues demonstrated that glioma CSCs release VEGF resulting in increased microvascular density in malignant glioma [168]. Monzani and colleagues also showed that melanoma CSCs co-expressed CD133 and VEGF [169]. Maeda and colleagues also showed that pancreatic CSCs co-express CD133 and VEGF-C resulting in increased microvascular density [170]. It is also possible that cancer cells may enter a state of dormancy in which they remain until induced to proliferate and form new tumors [171,172].

### 2.3. Cancer Stem Cells and Epithelial to Mesenchymal Transition

Besides the influence of genetic and epigenetic mechanisms on the CSC phenotype, the TME within which CSCs are located plays a huge role in the CSC behavior [30]. As more data emerges the CSC field continues to change and be refined [117,173]. Overall, the CSC phenotype is dynamic and never constant. When CSCs undergo EMT, they acquire characteristics allowing them to migrate, invade surrounding tissues and metastasize [174]. EMT and CSC characteristics appear to share similar molecular pathways that are involved in invasion and migration of cancer cells from the primary tumor. In addition, transcriptional analysis of EMT and those associated with CSCs reveal significant overlap in gene expression including TGF-β, Hedgehog signaling and microRNAs [26]. EMT has been associated with poor prognosis in several cancers including esophageal and colon cancers [175,176]. Several signaling pathways have been identified to be key in modulating CSCs behavior including invasiveness and metastatic ability [177,178]. In addition, several markers identifying CSCs with invasive and metastatic abilities have been revealed including CD44v6 [179,180]. CD44 is specifically expressed by breast epithelial cells undergoing EMT [181]. EMT is characterized by the loss of cell to cell adhesion with cells becoming mesenchymal and markers such as E-cadherin lacking in such cells [182,183]. The loss of E-cadherin from the cell surface is accompanied by the expression of N-cadherin [184]. Histone deacetylation of the CDH1 promoter through the actions of DNMT and HDACs leads to gene silencing [185,186]. Histone methylation within the CDH1 promoter via the EZH2 and PRC2 complex is known to silence its expression [187].

EMT is influenced by several protein factors as well as microRNAs. For example, TGF-β has been regarded as a master regulator of EMT in certain cancers including breast and colorectal cancers [188]. Besides influencing cancer cells, TGF-β can also regulate CAFs with a net effect of promoting metastasis [189]. Furthermore, microRNA-200 family members have been shown to suppress EMT via binding to two transcription factors, zinc finger E-box-binding homeobox 1 (ZEB1) and ZEB2 [190,191]. Tellez and colleagues demonstrated that EMT can be induced by epigenetic mechanisms including chromatin remodeling through H3K27me3 enrichment as well as DNA methylation to sustain silencing of tumor-suppressive microRNAs, microRNA-200b, microRNA-200c and microRNA-205 [192]. Thus, silencing these microRNAs through tri-methylation of DNMT and H3K27 can induce EMT-like and CSC characteristics [192].

### 2.4. Cancer Stem Cells and Metabolic Activity

Recently, metabolic alterations have been identified to cause cells to acquire stem-cell-like characteristics [193]. These alterations and the subsequent acquisition of stem-cell-like characteristics are thought to be caused by epigenetic changes in adult stem cells as well as cancer cells. Based on the CSC theory, acquisition of stem-cell-like characteristics makes these cells achieve a higher status within the hierarchy through the expression of self-renewal and pluripotent genes [30,78]. According to Menendez and Alarcon, products of mutated metabolic enzymes can behave as oncometabolites, inducing epigenetic changes in genetic material and thus drive tumor initiation and progression [193]. This and more pieces of evidence point to the need for a full view of tumor initiation and progression and not just focus on cancer cells. Metabolic processes can thus be targeted to stop tumor initiation and progression. Specifically, the TME is characterized by low oxygen and glucose levels and thus tends to favor oxidative phosphorylation as the main supplier of energy [194]. Hypoxia has been shown to induce metabolic alterations resulting in acidosis in several cancers [195,196]. Lee and colleagues demonstrated that chemoresistance and enhanced oxidative phosphorylation are correlated [194]. Recent studies demonstrated that indeed, the targeting of oxidative phosphorylation has shown some success in inhibiting CSCs metabolic processes and proliferation in some cancers [197,198].

Inhibition of the mitochondrial complex III resulted in decreased breast CSCs [199]. When relapse occurs, CSCs have been shown to increase oxidative phosphorylation levels to pretreatment levels, demonstrating the importance of oxidative phosphorylation in chemoresistance [200]. The adipose tissue and adipose-derived cells are able to interact with CSCs and have been shown to promote fatty acid oxidation in CSCs and chemoresistance [201]. The mitochondria are also known to play a role in CSC chemoresistance [202]. This is unsurprising as the mitochondria are central to many cellular processes such as metabolism, signaling and apoptosis. Mitochondria have recently been shown to play key roles in CSC behavior [203]. Sancho and colleagues concluded that the removal of CSCs through targeting mitochondrial function might prevent cancer disease from recurring and thus prevent fatal disease [204]. In colon CSCs, tumorigenic ability was associated with enhanced mitochondrial functions [205]. Atovaquone has been used to inhibit the mitochondrial complex II resulting in decreased breast CSCs [199]. Isayev and colleagues demonstrated that inhibition of glucose metabolism through the use of 3-bromopyruvate inhibited pancreatic CSCs growth and resistance to gemcitabine [206]. Several other studies also showed that inhibition of mitochondrial function affect CSC proliferation and self-renewal capabilities [207,208].

### 2.5. Cancer Stem Cells and Epigenetic Reprogramming

A contributing factor to the complex intra- and inter-tumor heterogeneity and the resulting failure of many anticancer therapies comes from CSC epigenetic alterations. The heritable non-genetic changes to CSCs phenotypes are what are called epigenetic reprogramming of CSCs [209,210]. Most of the proteins and enzymes involved in epigenetic reprogramming of cells including histone modifications and DNA methylations have been well-characterized [211,212]. For example, histone methyltransferases (HMTs) are responsible for methylation of histones whilst histone acetyltransferases are responsible for the acetylation of histones [213]. Demethylation and deacetylation of histones are carried out by histone demethylases (HDMs) and histone deacetylases (HDACs) respectively [213]. When acetylated, histones are more loosely packed and can be accessed by RNA polymerases, allowing transcription of genes around a specific location. On the other hand, methylation can activate or repress gene transcription. For example, the acetylation of histone H3/H4 is linked to the transcription of genes [214,215]. In addition, H3 lysine 4 methylation is also linked to transcription of several genes [216,217]. In contrast, the methylation of H3 lysine 9 and 27 is linked to gene repression [218,219,220]. It has been observed that different patterns of histone modification produce variable transcriptional outcomes, with some giving rise to activation of genes and others to repression [221,222]. Various mechanisms are known to be involved in epigenetic gene regulation, from modifications of cytosines in DNA, covalent modifications of histones, the involvement of noncoding RNAs to chromatin remodeling [223,224,225].

CpG islands are regions of the genome containing a large number of CpG dinucleotide repeats and usually extend for 300–3000 base pairs [226]. In most cases, CpG islands are located close to gene promoters in humans [227]. DNMT in addition to histone modification determines whether transcription occurs or not. When CpG islands are unmethylated, transcription can take place. When CpG islands are methylated the chromatin becomes transcription-suppressive. Methylation of CpG islands is catalyzed by DNMT1, DNMT3A and DNMT3B. Several tumor suppressor genes are silenced via CpG island methylation [228]. Transcription can also be repressed via the Polycomb repressive complexes 1 and 2 (PRC1 and PRC2) [229,230]. Polycomb repressors are able to catalyze the trimethylation of histone 3 lysine 27 (H3K27me3) giving rise to repression of genes associated with many cellular processes such as differentiation, development and choice of lineage [229,231]. Collinson and colleagues demonstrated that Polycomb complex PRC2 mediates H3K27me3 via the histone methyltransferase EZH2, leading to transcriptional repression of several genes [232].

CSCs and their subsets display epigenetic alterations including histone modifications and this eventually contributes to the intratumor heterogeneity observed in many tumors [233]. Several epigenetic regulators have mutations leading to tumor formation and progression as a result of epigenetic dysregulation [234,235]. Several CSC markers including CD133 are known to be regulated by epigenetic alterations [236]. Tabu and colleagues demonstrated that the hypomethylation of the CD133 promoter influences its expression in gliomas [237]. Yi and colleagues observed abnormal DNA methylation of CD133, a CSC marker, in colorectal and glioblastoma tumors [236]. Gorodetska and colleagues observed that EZH2/BRCA1 signaling mechanisms play an important role in the maintenance of prostate CSCs properties [238]. EMT aid in the generation of cells with stem cell characteristics and is modulated by epigenetic mechanisms [239,240]. The involvement of epigenetic mechanisms from CSC formation to maintenance makes epigenetics a therapeutic target in CSCs. Small compound inhibitors with the ability to induce differentiation in CSCs are therefore promising drugs targeting this population of tumor cells.

Several signaling pathways are crucial in facilitating the growth of CSCs and the maintenance of the CSC phenotype. Such signaling pathways include Hedgehog, Notch, JAK-STAT and Wnt-β- catenin signaling [241,242]. It is important to note that these same pathways are also important in regulating self-renewal in normal stem cells [243,244]. Several mutations have been observed in genes along these pathways in many human cancers. Signaling pathways such as Wnt and Notch have been observed in breast cancers for example [245] and in vitro work demonstrated that the overexpression of these pathways is associated with tumorigenicity [155,156]. Triple-negative breast cancer cells demonstrate increased Notch signaling and Notch signaling is associated with CD44 expression in colon cancer cells [158,246]. On the other hand, Wnt-β-catenin signaling has been observed to be associated with cancer stemness and heterogeneity [247]. Several members of the Wnt-β–catenin pathway have been linked to the induction of EMT in several cancers [248]. The hedgehog signaling pathway has been associated with self-renewal in many cancers including breast cancer and gliomas [249,250]. The hedgehog pathway has also been associated with EMT and invasion and migration [251,252].

Most of the above-mentioned signaling pathways are modulated by epigenetic mechanisms [253]. Under normal conditions, most of these pathways are involved in the propagation of CSCs, maintenance of the CSC phenotype as well as in embryonic development [254]. Several regulators of the above-mentioned pathways have been shown to have epigenetic alterations in CSCs. For example, decreased acetylation of H3K16 as well as enhanced H3K27 trimethylation is associated with DKK1 promoter silencing [255]. High levels of histone acetylation are observed at the promoter region of Notch receptor–ligand JAGGED2, resulting in Notch signaling activation in multiple myeloma cells [256]. In colorectal cancer, two Notch signaling targets, HES1 and HES2, show decreased promoter H3K27 methylation, resulting in gene activation [257,258,259]. Rhabdoid tumors show decreased or inactivation of SNF5, a member of chromatin remodeler complex SWI/SNF, leading to activation of Hedgehog signaling [260,261]. Furthermore, the activation of Gli1 and Gli2, downstream effectors of the Hedgehog signaling pathway, require HDAC1 [262,263,264]. As a result of the integration of genetic, epigenetic mechanisms and other factors, CSCs survival and maintenance are promoted.

The KMT2/MLL gene is known to encode for an HMT that influences many cellular processes [265,266]. MLL fusion proteins are present in several CSCs and have been shown to be involved in carcinogenesis in several cancers [267,268]. For example, Krivtsov and colleagues demonstrated that leukemia stem cells, with the MLL-AF9 fusion protein, can maintain the identity of progenitors from which they arose while at the same time activating stem-cell- or self-renewal-associated program [269]. Somervaille and colleagues also demonstrated that the hierarchical maintenance of MLL-myeloid leukemia stem cells utilizes a transcriptional program involving transcription/chromatin regulatory factors Myb, Hmgb3 and Cbx5 [270]. Several mutations have also been identified in histone-encoding genes. Lewis and colleagues showed that the blockage of PRC2 activity via the gain-of-function H3 mutation was prevalent in pediatric glioblastoma [271]. Furthermore, several DNMTs are mutated in acute myeloid leukemia and have been suggested to result in the formation of leukemia stem cells [272,273,274].

CSCs have been shown to play important roles in the propagation, growth and metastasis of colorectal cancer (CRC). Several genetic and epigenetic changes have been observed in CSCs in CRC. For example, the hypermethylation of several tumor suppressor gene promoters including p16, retinoblastoma, SFRP and MLH1, has been widely reported in many studies [7,275,276]. One of the driver mutations in CRC is the APC mutation, which influences the activities of DNMTs [277]. Increased levels of DNMT1 are thought to suppress the transcription of APC, a tumor suppressor gene in CRC [278,279]. The levels of DNMT1 in CSCs have been shown to be involved in CRC initiation and progression, directly linking epigenetic mechanisms to CSC-directed tumorigenesis [280]. Pathania and colleagues showed that DNMT1 is important for mammary and CSC maintenance and tumorigenesis [281].

## 3. Targeting Cancer Stem Cells in Tumor Microenvironment

Conventional anticancer therapies including chemotherapy and radiotherapy target rapidly proliferating cancer cells and can successfully debulk a tumor. However, several studies have shown that conventional therapies cannot prevent resistance, tumor relapse and metastasis [3,4]. It has been postulated that this is due to the presence of CSCs [30]. Several studies have shown that CSCs can easily undergo EMT, allowing these cells to promote tumor formation and progression [282,283]. The enhanced plasticity and heterogeneity observed within the CSC population, however, make targeting these cells daunting [196,284]. Currently, several strategies are employed to target CSCs in different cancers (Table 2). Combinations of surface markers have been used to isolate and characterize CSCs from different tissues. For example, CD44 and CD24 are used to isolate breast CSCs [285,286]. Combinations of drugs and antibodies have been used to target CSC surface markers successfully in different cancers [287,288]. In addition, the prevention of CSC surface markers from interacting with other proteins via the use of antibodies can result in CSCs being engulfed by immune cells, leading to tumor growth inhibition [289,290,291]. One such example is the use of a monoclonal antibody against CD47 named Hu5F9-G4 [292,293].

### 3.1. Targeting Cancer Stem Cell Signaling

Several CSC-specific signaling cascades have also been targeted in many cancers (Table 2). The Wnt-β-catenin has been observed to be dysregulated in CSCs in addition to several members of the pathway being mutated [294,295]. Several chemotherapeutic agents ranging from CWP232228, NCB-0846 and PRI-724 are either under clinical trial or being tested in in vitro research. PRI-724 targets CSCs by targeting their rapid cell division [296,297]. Jang and colleagues showed that CWP232228 preferentially targets breast CSCs in in vitro and animal cancer models [298,299].

Another important signaling cascade targeted in CSCs is the Notch pathway. Xu and colleagues demonstrated that inhibition of Notch signaling via the use of RO4929097 combined with chemotherapy has a beneficial effect in glioma patients with an observed reduction in CSCs [300]. Zhao and colleagues showed that chemotherapy together with another Notch inhibitor, DAPT (*N*-[*N*-(3, 5-difluorophenacetyl)-l-alanyl]-*S*-phenylglycine t-butyl ester), targeted CSCs in head and neck cancer [301]. Another signaling pathway that has been inhibited in CSCs is the Hedgehog pathway. The inhibition of the Hedgehog pathway through the use of nitidine chloride was shown to reduce CSC formation and abrogated the EMT process [302]. Hedgehog signaling inhibition coupled to the inhibition of the PI3K-Akt pathway was shown to reduce CSC self-renewal abilities [303]. Miyazaki and colleagues demonstrated that the combined inhibition of Hedgehog signaling and mTOR in pancreatic cancer cell lines suppressed CD133 expression and the ability of CSCs to form tumorspheres [304]. Clinical trials have been performed using gemcitabine and Smoothened inhibitor, Vismodegib [305,306].

Furthermore, the inhibition of the STAT3 pathway through the use of napabucasin was shown to reduce the viability of hematopoietic CSCs as well as their tumorigenic capabilities [307]. Napabucasin was also able to prevent relapse in pancreatic cancer after chemotherapy, demonstrating its effect on tumorigenic cancer cells [308]. Several clinical trials of napabucasin in combination with chemotherapy or immunotherapy are underway for the treatment of many cancers including colorectal carcinoma [309,310,311]. An inhibitor of the PI3K-Akt pathway, VS5584, has been shown to reduce CSCs in breast cancer and has shown its effectiveness at preventing relapse after chemotherapy [312,313]. Several therapeutic agents have been shown to affect many CSC signaling cascades and are therefore appealing. For example, salinomycin has been shown to selectively kill CSCs via inhibition of potassium flux as well as targeting the self-renewal properties of CSCs [314]. CSCs self-renewal is inhibited through the action of salinomycin on pathways such as Wnt and STAT3 signaling [181]. Salinomycin nanoparticles alone or in combination with chemotherapy when used on a breast cancer model were able to enhance mice survival [315].

### 3.2. Targeting Cancer-Stem-Cell-Associated Tumor Angiogenesis and Metastasis

Given the dependence of tumor formation and growth on the formation of blood vessels within the TME, inhibition of angiogenesis has been touted as having clinical value and could improve cancer patients’ outcomes (Table 2). One of the earliest anti-VEGF approved treatments involved the use of Bevacizumab, a monoclonal antibody that blocks the binding of VEGF to its receptor VEGFR [316]. Bevacizumab is mostly used together with commonly used drugs such as 5-fluorouracil as well as together with panitumumab [316,317]. Bevacizumab is currently being used for the treatment of colorectal, cervical and gastric adenocarcinoma [316,317,318,319]. Bevacizumab has not been successful in the treatment of other cancers such as breast cancer, with results showing poor patients’ overall survival [320,321]. Several tyrosine kinase inhibitors have been used to block angiogenesis including sunitinib and sorafenib. Importantly, sorafenib inhibits VEGFR in addition to PDGFR-β, thus can also affect the pro-tumorigenic behavior of stromal cells such as CAFs [322,323]. Sorafenib is currently being used to treat hepatocellular carcinoma, thyroid cancer and advanced renal cell carcinoma [324,325,326]. Sunitinib is also used for the treatment of renal cell carcinoma, thyroid cancer as well as advanced breast cancer [327,328,329,330]. Reports of resistance against antiangiogenic therapies have been published in addition to decreased amounts of therapy actually reaching cancer cells [331,332]. In addition, antiangiogenic therapies may increase CSCs through formation of hypoxic regions within tumors. Thus, it is important that thorough investigations are done for each drug to understand the mechanism of action.

The key to the formation of new blood vessels is the creation of space for cells to burrow through. Thus, matrix metalloproteinases (MMPs) and their inhibitors, the tissue inhibitors of metalloproteinases (TIMPs), play important roles in enabling the formation of new blood vessels [333,334,335]. Measured through the gold standard, which is the overall improvement of patients’ survival, MMPs inhibitors have produced disappointing results [334,336,337,338]. Many studies have shown that inhibition of MMPs has negative overall effects on normal cellular processes and thus detrimental to the human body [339,340,341]. Whilst MMPs are involved in tumor initiation and development, these enzymes are also important in normal cellular processes, making their inhibition a challenge. Selective and specific inhibitors of MMPs perceived to be involved in tumorigenesis have not been forthcoming or are still under investigation [342,343,344,345]. RO4929097, a gamma-secretase inhibitor, has been shown to reduce CSCs in glioma patients but the use of this inhibitor also resulted in the development of resistance [300,346]. One of the major disadvantages of inhibitors targeting signaling and enzymes (including MMPs) aberrantly expressed in CSCs is the negative side effects and the potential of therapy resistance. Several known protein factors such as cytokines and growth factors promote angiogenesis as well as the migration of CSCs [184,347,348]. Ginestier and colleagues demonstrated that blocking CXCR1 affects mostly breast CSCs in elaborate experiments involving the use of cells and xenografts [349]. The authors used Reparixin in elaborate experiments and showed that it has anti-CSCs activity in breast cancer cell lines [349]. Reparixin has been in several clinical trials with mixed results from such studies (Table 2) [350,351]. Further research involving blocking CXCR1/2 by Singh and colleagues also demonstrated decreased CSC activity in breast cancer [352]. Interactions between CSCs and the stromal component of the tumor microenvironment are mediated via chemokines and their receptors. For example, stromal-derived factor 1 and its receptor CXCR4 are both involved in the interactions between CSCs and cells such as CAFs and CAMs [347]. Stromal-derived factor has been implicated in cancer cell migration as well as invasion of nearby tissues for example [347]. Elaborate experiments by Gassenmaier and colleagues demonstrated that CXCR4 was upregulated in CSCs and that the inhibition of CXCR4 in renal cell carcinoma through the use of AMD3100 would hamper CSCs ability to proliferate and formation of tumorspheres [353]. As reviewed by Trautmann and colleagues, the use of combination therapy through targeting CXCR4-expressing CSCs in addition to radiotherapy can result in a durable cure for cancers [354].

### 3.3. Targeting the Immune System to Eradicate Cancer Stem Cells

Whilst research on the development of new drugs is an ongoing endeavor, new strategies being developed to eradicate cancer include targeting the stromal cells, CSCs and immune cells within the TME [4,30]. Importantly, the induction of an immune reaction to tumor cells as well as strengthening the immune system is some of the various methods being implemented (Table 2). Immunotherapy has been at the forefront of new strategies to boost the immune system of cancer patients, with the hope that it will lead to better patients’ outcomes. Several studies have demonstrated that the immune system can be used to fight cancer [355,356]. As immunotherapy works by inducing an immune response to cancer cells, it is possible to work for all cancers although results show varying patients response rates, with only a fraction of patients benefiting from such a treatment strategy [356]. Many candidate drugs that can inhibit the immune checkpoints are now in use or undergoing different levels of clinical trials [357,358,359,360]. Although several therapies have been developed, notable success came from antibodies targeting the programmed cell death 1 (PD-1) pathway alone or in combination with others [361,362,363]. PD-1 expression is triggered when the T cell receptor binds to cancer cells. In turn, PD-1 binds to PD-1 ligand (PD-L1) on cancer cells leading to exhaustion of T cells. Exhaustion of T cells dampens the anticancer cytotoxic T cell responses [364]. Another promising therapy involves the use of antibodies against the cytotoxic T lymphocyte-associated protein 4 (CTLA-4) [365,366,367]. CTLA-4 causes T cell inhibition via competing with stimulatory molecules for T cells. Binding of CTLA-4 to receptors on T cells causes inhibition of T cell proliferation, dampening cancer cell recognition and killing [368]. By blocking this immune checkpoint through the use of antibodies, allows T cells to proliferate and be able to recognize antigens on the cancer cell surface.

Several antibodies have been developed to induce an anticancer immune response. For example, Ipilimumab is an anti-CTLA-4 inhibitor that was developed and used in patients with advanced melanoma [369]. Patients displayed improved and durable responses but side effects including inflammation of endocrine glands were observed. In combination with others such as Nivolumab, Ipilimumab has been used for several cancers including renal cell carcinoma, melanoma, metastatic colorectal cancer, small cell lung cancer and metastatic esophagogastric cancer [369,370,371]. Promising results from these antibodies led to the approval of several others including avelumab, pembrolizumab, durvalumab and atezolizumab [372,373,374,375]. When used in different cancers, these checkpoint inhibitors display varying response rates with some showing very high responses such as in Hodgkin’s disease where the response rate was around 90 percent [376,377,378]. Reports of cardiotoxicity and pneumonitis show that further research is still required to reduce the side effects associated with these antibodies [379,380]. Interestingly some reports show that checkpoint inhibitors may work synergistically with antibodies against other markers such as HER2 in breast cancer [381,382,383,384].

Resistance to immune checkpoint therapy has been suggested to be caused by CSCs. Stemness as well as increased angiogenesis has been associated with reduced recognition of T cells [385,386,387]. Several studies have demonstrated that CSCs have the ability to evade the immune system [387,388]. Wu and colleagues showed that the overexpression of PD-1 may be the reason CSCs are able to evade the immune system [389]. Bruttel and Wischhusen on the other hand showed that CSCs evade the immune system via lack of molecules needed for T cell recognition [390]. Several other studies showed that CSCs evade the immune system due to their creation of an immune suppressive microenvironment [391,392]. Despite the above, the high expression of PD-L1 on the CSCs’s surfaces makes CSCs targets of checkpoint inhibitors. Standard therapies can be applied first followed by immunotherapy to wipe out the remaining CSCs [393,394,395].

One of the best immunotherapies under trial and available for cancer patients is the chimeric antigen receptor (CAR) T cell transfer. CAR T cell transfer can be used for both solid and liquid malignancies [396,397]. CAR T cells can potentially identify any marker or antigen on the surface of CSCs and thus makes them an appealing substrate for the development of CSC-specific therapies. As reviewed by Guo and colleagues, CAR T cells offer a curable approach for the treatment of cancer and the avoidance of fatal disease [151]. Several studies have investigated the use of CAR T cells together with standard and other therapies for the treatment of different cancer types. For example, Feng and colleagues investigated the potential of combining two CAR T therapies in patients with advanced Cholangiocarcinoma (CCA) [398]. The authors observed that CAR T therapy may be feasible for the treatment of Cholangiocarcinoma (CCA) but cautioned its use before further studies were done due to possible toxicities [398]. In their study, the authors used CAR T anti-EGFR and anti-CD133 in order to specifically target CSCs [398]. Several clinical trials utilizing CAR T therapy are underway for different cancers. Both CAR T anti-EGFR and anti-CD133 are under clinical trials (NCT02541370 and NCT01869166). In another study, Guo and colleagues observed that CAR T anti-EGFR cell immunotherapy was a safe way to treat EGFR-positive advanced biliary tract cancers [399]. In yet another study, a combination of haploidentical CD19-CAR T cells and stem cells achieved full donor engraftment in refractory acute lymphoblastic leukemia [400]. Utilizing well-characterized CSC markers it is possible therefore to use CAR T cells to eliminate CSCs in many cancers. The use of CAR T cells can also solve the problem of non-universal expression of some markers. Whether used alone or in combination with checkpoint inhibitors or standard therapy, CAR T cells are a promising strategy for the treatment of many cancers. As CAR T cells and also checkpoint inhibitors target markers on CSCs and the immune system respectively, both treatment strategies can result in improved treatment outcomes by not being targeted at specific cancer cells.

The above-described strategies require moderation as the overstimulation of the immune system can be detrimental. Done properly with proper control, immunotherapy can become a very good and natural way to respond to the presence of cancer cells in the body. Challenges remain in terms of immunotherapy for cancer treatment. For example, Noh and colleagues demonstrated that CTL-mediated immune selection drives tumor cell evolution toward the CSC phenotype, with the resulting CSCs demonstrating great heterogeneity [401].

### 3.4. Targeting Epigenetic Modifications in Cancer Stem Cells

Recent data point to possible manipulation of epigenetic states or mechanisms in cancer cells by altering molecular factors that are involved. Major hurdles remain including the identification of compounds and agents able to selectively target epigenetic mechanisms in cancer cells at low concentrations. HDAC inhibitors for example are mostly considered as pan-inhibitors and display many side effects. Many pan-HDAC inhibitors have been approved by the FDA or are under trials. One well studied HDAC inhibitor is Vorinostat, which targets HDAC-1-3 and HDAC 6. Several clinical trials using Vorinostat as a standalone drug or in combination with others are underway for cancers that relapsed or other solid tumors [402,403,404]. Other HDAC inhibitors under clinical trial include Romidepsin which is being studied for both pediatric and adult cancers [405,406]. In addition, DNMTs inhibitors including Azacitidine and Decitabine are also under different stages of clinical trial for several cancers.

The BET family, which are chromatin readers, interact with chromatin modifiers as well as enzymes to affect chromatin modification. Proteins containing bromodomains dock on acetylated histones [407,408]. Consequently, the histone code will influence not only the DNA sequence but also the transcription factors involved [153]. BET inhibitors including JQ1 and I-BET762 have shown efficacy in clinical trials against CSCs in several cancers such as neuroblastoma, acute myeloid leukemia and NUT midline carcinoma [409,410,411]. Major hurdles remain on the use of BET inhibitors with side effects including toxicity to normal cells development of resistance [412,413]. To overcome possible resistance to standard therapy and treatment with epigenetic drugs including BET inhibitors, combination therapy is usually done during treatment. Chemotherapy and radiotherapy can be combined with pan-HDAC and BET inhibitors or with immunotherapy for durable cancer treatment. In most cases, combining epigenetic drugs such as BET inhibitors with chemotherapy and immunotherapy demonstrate synergistic effects in both cells and animal models [414,415]. Li and colleagues demonstrated that drugs targeting cancer cell epigenetics potentiate chemotherapy effects in solid tumors [416,417]. HDAC inhibitors and DNMTs inhibitors have been shown to have synergistic effects in leukemia cells [418,419].

## 4. Conclusions

Great improvements and success in cancer treatments has been recorded in the past few years mainly due to prevention campaigns, early diagnosis and better therapies. Overall, better and improved cancer patient outcomes have been observed. Even with these observations, millions of cancer patients die each year. One major hurdle to improved cancer patient outcomes is the development of resistance to therapies and disease relapse [3]. Important in cancer relapse is the presence of CSCs, a subpopulation of cancer cells with self-renewal and tumorigenic properties. Whilst many studies and drugs still target all cancer cells within a tumor in order to debulk the tumor, more research is targeted against CSCs. The identification and characterization of CSCs within different tumors can reveal their characteristics and markers that can be used in their elimination. To this end several markers including CD44, ALDH1 and CD133 have been identified in different cancers. Novel strategies including the use of nanotechnology aim to detect, characterize and eliminate CSCs with enhanced efficacy than current methods [420,421]. Currently, several chemotherapeutic agents targeting CSCs are under investigation and some included in clinical trials. CSCs being a small subpopulation of cancer cells may prove to be easily eradicated if targeted properly. Combining CSC surface marker targeting using drugs loaded onto nanomaterials can effectively be used against CSCs [422,423]. Ligands of CSC surface markers allow increased specificity in terms of targeting CSCs and have already been shown to be effective [5,424]. Further research into the solubility and other features of these carriers is underway and is likely to yield better molecules leading to better treatments. To this end, the use of cancer organoids as models of tumors may aid in preclinical studies [30,34]. These new treatment strategies must target the different strategies used by CSCs to survive and promote cancer relapse such as activation of survival signaling pathways, immunosuppression and enhanced metabolic adaptation.

## Figures and Tables

**Table 1:** CSC markers expressed in different human cancers *.

Cancer	CSC Markers	References
Cervical	CD133+, CD49f+, CK-17+	[35,36,37]
Esophageal	CD44+, ALDH1+, Integrin α7+	[38,39]
Kidney	CD24-, CD44+, CD105+, CD133+	[40,41,42]
Lung cancer	CD44+, CD90+, CD133+, ABCG2+, ALDH+	[24,33]
Colon cancer	CD24+, CD44+, CD133+, EpCAM+, ALDH+	[43,44,45,46]
Liver cancer	CD24+, CD44+, CD90+, CD133+, ALDH+, ABCG2+	[47,48]
Breast cancer	CD24-, CD44+, CD133+, ALDH-1+	[5,49,50]
Gastric	CD44+, CD133+	[51,52,53]
Glioma	CD44+, CD133+, A2B5+, BCRP1+, SSEA-1+	[54,55]
Leukemia (AML)	CD34+, CD38−, CD123+	[56,57,58]
Leukemia (CML)	CD25+, CD26+, CD44+, CD93+, IL1RAP+	[59,60]
Ovarian	CD44+, CD117+, CD133+, ALDH1+	[61,62]
Prostate cancer	CD44+, CD133+, α2β1+, ALDH+	[63,64,65]
Pancreatic cancer	CD44+, CD133+, ABCG2+, ALDH+, EpCAM+	[66,67,68]
Melanoma	ABCB5+, CD20+	[69,70]
Head and neck cancer	CD44+, CD133+	[71,72]
Sarcoma	CD29+, CD117+, CD133+, Nestin+, Stro-1+	[73,74]

* The list of CSC markers is not exhaustive. The CSC markers continue to be refined based on new data.

**Table 2:** Drugs currently under trial in combination with chemotherapy and radiotherapy for the treatment of different cancers.

Cancer Type	Chemotherapy/Radiotherapy/Immunotherapy	Clinical Trial Identifier
Breast	Ruxolitinib + Chemotherapy	NCT02876302
	Lapatinib + Radiotherapy	NCT01868503
	Paclitaxel + Reparixin	NCT02370238
	Paclitaxel + Reparixin	NCT02001974
	Vorinostat + Lapatinib	NCT01118975
	MK-0752 + Docetaxel + Pegfilgrastim	NCT00645333
Colorectal	OMP-305B83 + FOLFIRI + FOLFOX	NCT03035253
	Napabucasin + Fluorouracil + Leucovorin + Irinotecan + Bevacizumab	NCT02753127
	OMP-21M18	NCT01189942
Esophageal	Dietary Supplement: Fursultiamine	NCT02423811
Gastrointestinal	Phase 1: BBI608 Phase 2: Fluorouracil + Oxaliplatin + Leucovorin + Irinotecan + Bevacizumab + Capecitabine + Regorafenib	NCT02024607
Glioma	3-Dimensional Conformal Radiation Therapy + Gamma-Secretase Inhibitor RO4929097 + Intensity-Modulated Radiation Therapy + Temozolomide	NCT01119599
	ChemoID assay + Chemotherapy	NCT03632135
	Stem Cell Radiotherapy (ScRT) + Temozolomide	NCT02039778
Head and Neck	IPI-926 + Cetuximab	NCT01255800
Hematologic	Azacitidine + SL-401 + Venetoclax	NCT03113643
	Lenalidomide + Dexamethasone + MEDI-551	NCT01861340
	Zileuton	NCT01130688
Hepatocellular	BBI608 + BBI503 + Sorafenib	NCT02279719
	Metformin	NCT01442870
Ovarian	Chemotherapy	NCT03632798
	Carboplatin + Paclitaxel + Ruxolitinib + Ruxolitinib Phosphate	NCT02713386
	Metformin	NCT01579812
Pancreatic	Gamma-secretase/Notch signaling pathway inhibitor RO4929097	NCT01192763
	Demcizumab + Abraxane^®^ + Gemcitabine	NCT01189929
	Cyberknife radiation + gemcitabine	NCT01051284

## Abbreviations

ABC	ATP-binding cassette
ALDH1	aldehyde dehydrogenase 1
ATRA	All-trans retinoic acid
BMI1	B-cell-specific Moloney murine leukemia virus integration site 1
BMP-4	Bone morphogenic protein 4
CAR T	Chimeric antigen receptor
CCA	Cholangiocarcinoma
CK	cytokeratin
CNS	central nervous system
CRC	Colorectal Cancer
CSCs	Cancer stem cells
CTL	Cytotoxic T lymphocyte
DCLK1	Double cortin-like kinase 1;
EMA	epithelial membrane antigen;
EMT	Epithelial to mesenchymal transition
EpCAM	epithelial cell adhesion molecule;
GCSF	Granulocyte colony stimulation factor
H3K27me3	Histone 3 lysine 27
HDACs	Histone deacetylases
HDMs	Histone demethylase
HMTs	Histone methyltransferase
HNSCC	head and neck squamous cell carcinoma
K17	Keratin 17
Lgr5	Leucine-rich repeat-containing G-protein coupled receptor 5
MDR1	Multidrug resistance 1
MGMT	O6-methylguanine DNA methyltransferase
MMPs	Matrix metalloproteases
PD-1	Programmed cell death 1
PD-L1	PD-1 ligand
PSA	prostate-specific antigen
TME	Tumor microenvironment
TIMPs	Tissue inhibitors of metalloproteinases
TMZ	Temozolomide
ZEB1	Zinc finger E-box-binding homeobox 1
